# Composition and Biological Activity of the Essential Oils from Wild Horsemint, Yarrow, and Yampah from Subalpine Meadows in Southwestern Montana: Immunomodulatory Activity of Dillapiole

**DOI:** 10.3390/plants12142643

**Published:** 2023-07-14

**Authors:** Igor A. Schepetkin, Gulmira Özek, Temel Özek, Liliya N. Kirpotina, Robyn A. Klein, Andrei I. Khlebnikov, Mark T. Quinn

**Affiliations:** 1 Department of Microbiology and Cell Biology, Montana State University, Bozeman, MT 59717, USA; igor@montana.edu (I.A.S.); liliya@montana.edu (L.N.K.); 2 Department of Pharmacognosy, Faculty of Pharmacy, Anadolu University, Eskisehir 26470, Turkey; gulmiraozek@gmail.com (G.Ö.); temelozek@gmail.com (T.Ö.); 3 Department of Plant Sciences and Plant Pathology, Montana State University, Bozeman, MT 59717, USA; herbrobin@gmail.com; 4 Kizhner Research Center, Tomsk Polytechnic University, 634050 Tomsk, Russia; aikhl@chem.org.ru

**Keywords:** *Agastache urticifolia*, *Achillea millefolium*, *Perideridia gairdneri*, dillapiole, chemotaxis, essential oil, neutrophil

## Abstract

*Agastache urticifolia* (Benth.) Kuntze (horsemint), *Achillea millefolium* L. (yarrow), and *Perideridia gairdneri* (Hook. & Arn.) Mathias (yampah) are native, culturally important plants that grow in the subalpine meadows of Montana. Analysis of the composition of essential oils extracted from these plants showed that the main components of essential oils obtained from flowers and leaves of *A. urticifolia* (designated as AUF/AUL) were menthone (2.7/25.7%), isomenthone (2.6/29.1%), pulegone (78.9/28.8%), and limonene (4.2/6.2%), whereas essential oils obtained from the inflorescence of *A. millefolium* (designated as AMI) were high in α-thujone (17.1%) and β-thujone (14.9%), 1,8-cineole (17.0%), camphor (13.0%), sabinene (7.0%), guaia-3,9-dien-11-ol (3.2%), and terpinen-4-ol (2.5%). Essential oils obtained from the inflorescence of *P. gairdneri* (designated as PGI) contained high amounts of dillapiole (30.3%), *p*-cymen-8-ol (14.1%), terpinolene (12.0%), 4-hydroxy-4-methyl-cyclohex-2-enone (6.2%), and γ-terpinene (2.4%). Evaluation of their immunomodulatory activity demonstrated that essential oils extracted from all of these plants could activate human neutrophils with varying efficacy. Analysis of individual components showed that dillapiole activated human neutrophil intracellular Ca^2+^ flux ([Ca^2+^]_i_) (EC_50_ = 19.3 ± 1.4 μM), while α-thujone, β-thujone, menthone, isomenthone, and pulegone were inactive. Since dillapiole activated neutrophils, we also evaluated if it was able to down-regulate neutrophil responses to subsequent agonist activation and found that pretreatment with dillapiole inhibited neutrophil activation by the chemoattractant *f*MLF (IC_50_ = 34.3 ± 2.1 μM). Pretreatment with *P. gairdneri* essential oil or dillapiole also inhibited neutrophil chemotaxis induced by *f*MLF, suggesting these treatments could down-regulate human neutrophil responses to inflammatory chemoattractants. Thus, dillapiole may be a novel modulator of human neutrophil function.

## 1. Introduction

*Agastache urticifolia* (Benth.) Kuntze (horsemint), *Achillea millefolium* L. (yarrow), and *Perideridia gairdneri* (Hook. & Arn.) Mathias (yampah) are native, culturally important plants that can be found in the subalpine meadows of Montana. The leaves of all three species are strongly aromatic, especially when crushed. Analysis of ethnobotanical reports recorded in the Native American Ethnobotany database indicated that a decoction of *A. urticifolia* leaves was used for rheumatism and rhinitis [1]. Likewise, the Cheyenne people have used roots, stems, and leaves of *P. gairdneri* for different medicinal purposes [1], Blackfoot people have used roots of *P. gairdneri* to draw inflammation from swellings and as a nostril wash for rhinitis [1], and the Cherokee people have used *A. millefolium* for treating bloody hemorrhoids, bloody urine, and bowel complaints [1,2]. *Achillea millefolium* L. has also been widely used as a wound-healing agent and to treat gastrointestinal complaints [2,3], and infusions of the herb have been used as a treatment for fever [1,2]. Lastly, yarrow extract has been reported to exhibit spasmogenic effects in murine and human gastric antrum [4].

Essential oils are one of the bioactive components present in medicinal plant extracts and are currently recognized for their medicinal properties. For example, essential oils have been reported to exhibit immunomodulatory and anti-inflammatory effects [5,6,7]. Some of the earliest innate immune cell types that respond to the presence of pathogenic organisms are neutrophils [8]. Neutrophils are recruited to inflammatory sites of injury or infection by a variety of factors, including *N*-formyl-Met-Leu-Phe (*f*MLF), a bacterial or mitochondria-derived peptide, and chemokines [9]. Chemokines activate neutrophil G-protein coupled receptors (GPCRs) and stimulate chemotaxis, as well as the production of inflammatory mediators, including reactive oxygen species, cytokines, and proteases [9]. We recently found that essential oils from *Populus balsamifera*, *Grindelia squarrosa*, *Rhododendron albiflorum*, *Juniperus,* and *Artemisia* spp. can modulate human neutrophil functions [10,11,12,13,14]. Likewise, essential oils of *A. millefolium* have been clinically recognized as a treatment for wounds and other skin-inflammatory conditions [15]. In contrast, not much is known about the therapeutic properties of the essential oils from the other two plant species studied in this research, *A. urticifolia*, and *P. gairdneri* [16,17,18].

Based on the reported therapeutic effects of extracts of horsemint, yarrow, and yampah, this work aimed to evaluate the composition and innate immunomodulatory activity of essential oils from these plants. Essential oils were isolated from these plants and analyzed for their chemical compositions and innate immunomodulatory activities. Interestingly, these essential oils exhibited immunomodulatory activity and inhibited intracellular Ca^2+^ mobilization ([Ca^2+^]_i_) in activated human neutrophils. Furthermore, dillapiole, which was present at high levels in essential oils of *P. gairdneri* also inhibited human neutrophil functional responses. Thus, dillapiole is likely one of the main active components in these essential oils. Since neutrophils play an important role in inflammation, these data suggest that dillapiole could be considered in the development of new anti-inflammatory agents.

## 2. Results and Discussion

### 2.1. Plant Material

Plant material was collected in July 2021 near Bozeman, MT, USA (Table 1). The plant material was air-dried at room temperature for 7–10 days in the dark before hydrodistillation. Botanical identification of the plant material was performed at Montana State University, Bozeman, MT, USA.

### 2.2. Essential Oil Composition

The distillation yields (*v*/*w*) of essential oils obtained from the three plant species were 0.2 to 1.9% (Table 1). Simultaneous GC-FID and GC/MS were used to evaluate the chemical composition of these essential oils (Table 2), and a summary of their chemical composition is shown in Table 3. A total of 55/44, 65, and 43 compounds, accounting for 97.4%/98.5%, 98.7%, and 80.0% of the essential oils from flowers and leaves of *A. urticifolia* (designated as AUF/AUL), inflorescences of *A. millefolium* (designated as AMI), and inflorescences of *P. gairdneri* (designated as PGI) respectively, were identified and quantified.

Major compounds of AUF were pulegone (78.9%), limonene (4.2%), menthone (2.7%), isomenthone (2.6%), piperitenone (1.6%), and *trans*-*p*-mentha-8-methylthio-3-one (1.1%). Similarly, the major compounds of AUL were isomenthone (29.1%), pulegone (28.8%), menthone (25.7%), and limonene (6.2%). Thus, these results are consistent with previous findings suggesting that *A. urticifolia* essential oils are primarily composed of limonene, menthone, and pulegone, although isomenthone was found as a minor compound in native Oregon and Utah, USA plant populations [17]. Biological characteristics and dynamics of essential oil content of *A. urticifolia* in Moldova have also been reported [16]. In the essential oil of *A. urticifolia* from Moldova, 17 compounds were identified, with the basic ones being phenylpropanoids, estragole (41.1%) and methyl eugenol (5.1%), as well as monoterpenes, pulegone (20.4%), limonene (15.3%), isomenthone (12.0%), and menthone (1.7%) [19]. The essential oils of *A. urticifolia* cultivated in the Middle Ural (Russia) contained a high abundance of monoterpenes, including menthone (23.0%), isomenthone (9.9%), and pulegone (5.6%). Sesquiterpenes were also present, including spathulenol (5.4%), α-cadinol (1.8%), and caryophyllene-4(12)8(13)-diene-5α-ol (1.5%) [20]. In general, a literature survey and comparative evaluation of *Agastache* profiles revealed that the composition of essential oils is relatively variable, but with phenylpropanoids and oxygenated monoterpenes predominating. Namely, estragole (*syn*. methylchavicol), methyleugenol, and (*E*)-anethole are usually the most abundant constituents. Other chemotypes of *Agastache* are rich in menthone, isomenthone, pulegone, and limonene [18].

We also found sulfur-containing monoterpenes [*trans*-*p*-mentha-8-methyl-thio-3-one (1.1% and 0.8%) and *cis*-*p*-mentha-8-methyl-thio-3-one (0.5% and 0.4%)], in flower and leaf essential oils of *A. urticifolia*. The thio-compounds are perhaps responsible for the characteristic scent of these oils. Notably, this is the first report of thio-monoterpenes in *Agastache* essential oils. Previously, different representatives of the Lamiaceae family, e.g., *Agathosma* and *Calamintha* species, have been reported to contain sulfur-monoterpenes [21,22]. Likewise, a sulfur derivative of pulegone was reported to be a major constituent of buchu (*Agathosma betulina*) essential oils, as well as methylthio- and acetylthio-derivatives of pulegone and other *p*-menthane constituents [23].

Essential oils from inflorescences of *A. millefolium* (AMI) contained high amounts of α-thujone (17.1%), β-thujone (14.9%), 1,8-cineole (17.0), camphor (13.0%), sabinene (7.0%), guaia-3,9-dien-11-ol (3.2%), and terpinen-4-ol (2.5%). High amounts of α-thujone and β-thujone were previously reported in essential oils extracted from *A. millefolium* collected in Europe and Chile [24,25,26]. High levels of oxygenated monoterpenes (53.9–76.1%), mainly α- and β-thujone (up to 26.8%), camphor (up to 24.5%), 1,8-cineole (up to 20.3%) and artemisia ketone (up to 10.1%), were identified in essential oils of *A. millefolium* from France, Belgium, Spain, Italy, Russia, and Armenia. The content of chamazulene in these samples was only 0–0.8%. In the literature, relatively high amounts of the above-mentioned terpenes were reported to be typical for hexaploid yarrow plants. Additionally, 1,8-cineole and camphor were primary components of *A. millefolium* essential oils from Serbia, France, and Eastern Turkey [27,28,29]. According to “Millefolii Herba” from the European Pharmacopoeia, the content of proazulenes expressed as chamazulene should be a minimum of 0.02% (dried drug) in yarrow [30]. However, proazulenes were not detected in our samples. A literature survey revealed that yarrow essential oils from Chile were rich in β-thujone (96.2%), while other compounds identified were α-thujone, 1,8-cineole, *p*-cymene, and 4-terpineol (all < 1.0%) [26]. Significant variations in essential oil content and composition in commercial samples of yarrow were reported by Raal et al. [31], with the most important components of yarrow essential oils being chamazulene (0.8–44.3%), β-pinene (tr—23.3%), sabinene (0–16.5%), bornyl acetate (tr—15.8%), (*E*)-β-caryophyllene (2.5–14.3%), (*E*)-nerolidol (tr—9.6%), 1,8-cineole (trace—9.6%), and germacrene D (0.2–7.8%). Chemotypes containing chamazulene, chamazulene + bornyl acetate, chamazulene + β-pinene + (*E*)-β-caryophyllene, sabinene + 1,8-cineole, and β-pinene + α-terpinyl acetate have also been reported [31]. Such variation in the composition of yarrow essential oils may be due to various factors related to chemotype, ecotype, phenophases, altitude, and variations in environmental conditions, such as temperature, photoperiod, relative humidity, and irradiance. Moreover, genetic background may be the factor responsible for affecting the chemistry of secondary metabolites of these plants. The chemical composition also varies strongly due to different ploidy (di-, tetra-, hexa-, octoploid), and frequent hybridization within this group but also with different *Achillea* species. For example, the major constituents of tetraploid *A. millefolium* plants include chamazulene, β-pinene, and caryophyllene, while octoploid plants contain ~80% oxygen-containing monoterpenes, with linalool as the major constituent [32].

Essential oils from inflorescences of *P. gairdneri* (PGI) contained high amounts of dillapiole (30.3%), *p*-cymen-8-ol (14.1%), terpinolene (12.0%), 4-hydroxy-4-methyl-cyclohex-2-enone (6.2%), and γ-terpinene (2.4%). This is the first report on the composition of essential oils extracted from *P. gairdneri*. The high content of 4-hydroxy-4-methyl-cyclohex-2-enone is interesting since this compound was reported in flower essential oils from *Hypericum perforatum* [33] and *Ledum palustre* var. *nipponicum* [34] and can be metabolized to pulegone [35].

### 2.3. Effect of Essential Oils and Selected Component Compounds on Neutrophil Ca^2+^ Influx

**Figure 1:**
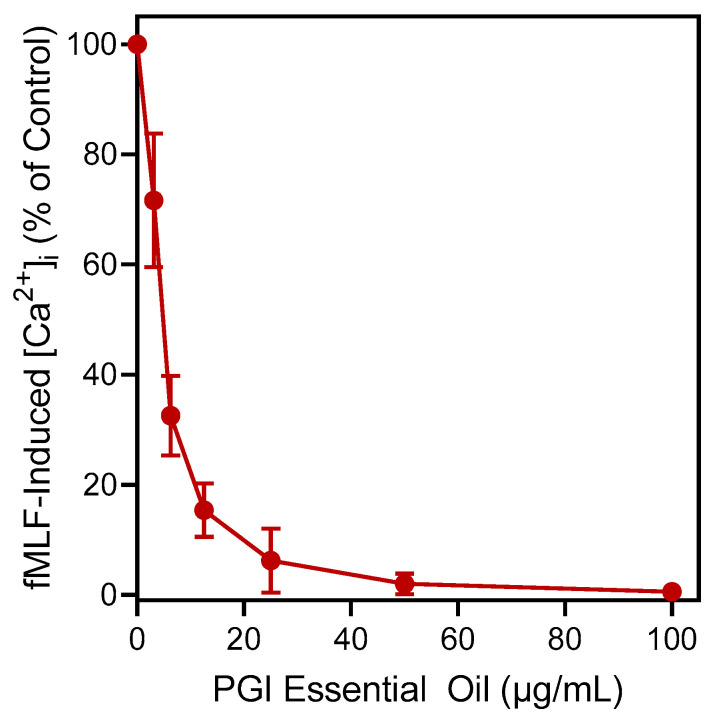
Effect of the PGI essential oil on *f*MLF-induced neutrophil [Ca^2+^]_i_. Human neutrophils were incubated with the indicated concentrations of the essential oil or 1% DMSO (negative control) for 10 min. The cells were then activated with 5 nM *f*MLF, and [Ca^2+^]_i_ was monitored as described. The data shown are presented as the mean ± SD from one experiment that is representative of three independent experiments with similar results.

We evaluated the essential oils for their immunomodulatory effects on human neutrophils. In particular, the effects of the essential oils on intracellular Ca^2+^ flux ([Ca^2+^]_i_) were assessed, since [Ca^2+^]_i_ is an important signal during neutrophil activation. Treatment of neutrophils with essential oils from *A. urticifolia* (AUF and AUL), *A. millefolium* (AMI), and *P. gairdneri* (PGI) activated human neutrophils, resulting in increased [Ca^2+^]_i_, with EC_50_ values ranging from 28.5 to 43.5 µg/mL (Table 4). Pre-incubation of neutrophils with the most active of these essential oil samples (PGI) inhibited the subsequent neutrophil [Ca^2+^]_i_ response to the chemoattractant *f*MLF with an IC_50_ of 4.3 µg/mL (Figure 1), while other essential oil samples had lower inhibitory activity (Table 4).

Previously, several of the compounds that are present in the essential oils evaluated here were shown to have no activation and (or) inhibitory effects on human neutrophil [Ca^2+^]_i_, including camphor, 1,8-cineole, *p*-cymene, *p*-cymen-8-ol, elemicine, hexanal, limonene, linalool, myrcene, (*E*/*Z*)-β-ocimene, β-phellandrene, α-pinene, β-pinene, piperitenone, sabinene, spathulenol, α-terpinene, terpinen-4-ol, and terpinolene [12,13,36,37]. In contrast, (±)-bornyl acetate, (−)-borneol, germacrene D, and nerolidol were found previously to inhibit agonist-induced activation of human neutrophils [10,11,12,13,33]. Thus, the inhibitory effects of AMI essential oils on human neutrophil Ca^2+^ flux are likely due to the presence of bornyl acetate, (−)-borneol, germacrene D, and nerolidol, whereas germacrene D and some other minor components could be responsible for the biological activity of AUF/AUL.

**Figure 2:**
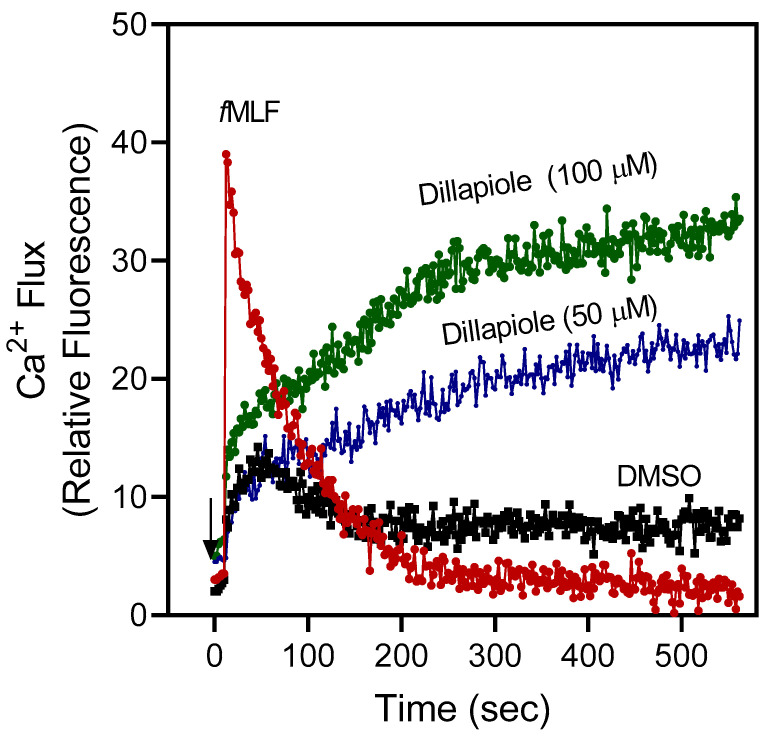
Effect of dillapiole on neutrophil [Ca^2+^]_i_. Human neutrophils were treated with dillapiole (50 and 100 µM), 5 nM *f*MLF (positive control), or 1% DMSO (negative control), and [Ca^2+^]_i_ was monitored for the indicated times (arrow indicates when treatment was added). Data are from one experiment that is representative of three independent experiments.

We evaluated the activity of additional constituent compounds from our essential oil samples that have not been evaluated previously in human neutrophils, including α-thujene, α/β-thujone, menthone, isomenthone, pulegone, and dillapiole. The results showed that only dillapiole, a major component of PGI, was active (Table 4, Figure 2). Indeed, dillapiole effectively activated human neutrophil [Ca^2+^]_i,_ with an EC_50_ of 19.3 μM. Note that the addition of control *f*MLF caused a rapid increase in [Ca^2+^]_i_ that peaked by 1 min and gradually declined to basal values, reflecting the rapid clearance of Ca^2+^ from the cytosol. The time course of [Ca^2+^]_i_ induced by dillapiole is different from that observed in *f*MLF-stimulated cells and likely reflects activation of a different pathway in neutrophils. Further studies will be important to define the specific receptor or target of dillapiole.

**Figure 3:**
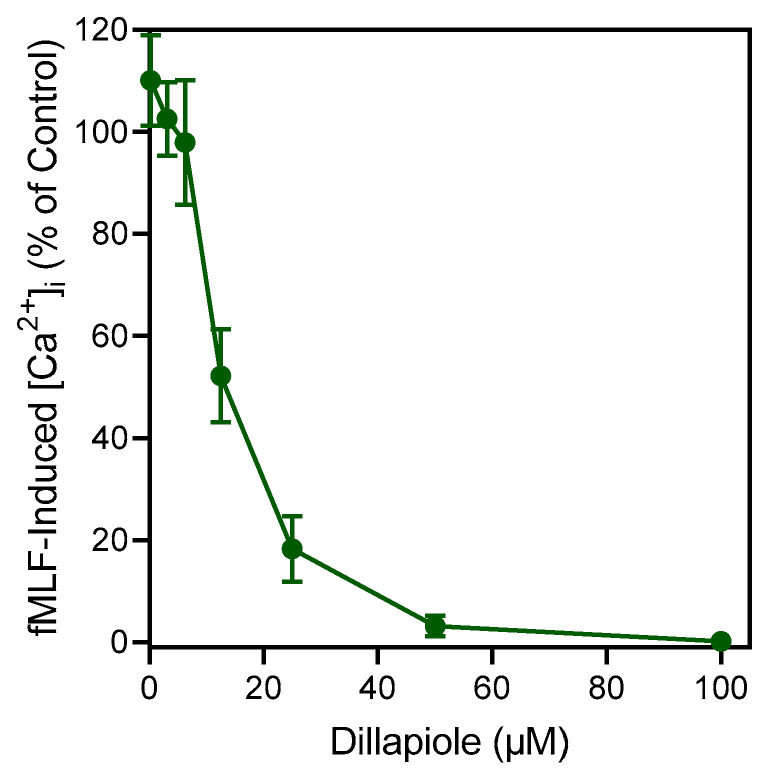
Effect of dillapiole on *f*MLF-induced neutrophil [Ca^2+^]_i_. Human neutrophils were treated with the indicated concentrations of the dillapiole or 1% DMSO (negative control) for 10 min. The cells were then activated with 5 nM *f*MLF, and [Ca^2+^]_i_ was monitored as described. The data shown are presented as the mean ± SD from one experiment that is representative of three independent experiments with similar results.

Since dillapiole directly activated neutrophil [Ca^2+^]_i_, albeit with low efficacy, it is possible that this compound could contribute to receptor desensitization and/or intracellular Ca^2+^ store depletion. Indeed, pre-incubation of neutrophils with dillapiole inhibited subsequent *f*MLF-induced [Ca^2+^]_i_, with an IC_50_ of 13.9 μM (Figure 3). Note that essential oils from *A. urticifolia* contained predominantly the (*S*)-(−) enantiomer of pulegone [17]. Here, we evaluated the activity of commercially available (*R*)-(+)-pulegone. Thus, we cannot exclude an activity of (*S*)-(−)-pulegone in human neutrophils since that isomer is not commercially available.

### 2.4. Effect of PGI Essential Oil and Dillapiole on Neutrophil Chemotaxis

**Figure 4:**
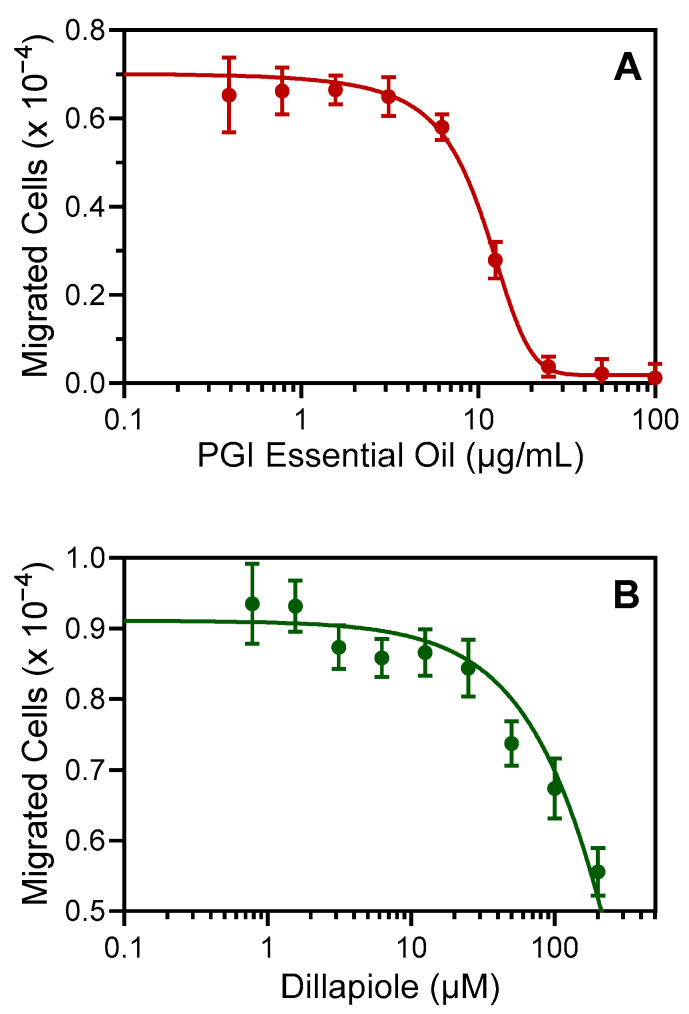
Effect of the PGI essential oil and dillapiole on human neutrophil chemotaxis. Neutrophils were pretreated with the indicated concentrations of the essential oil (**A**) or dillapiole (**B**), and neutrophil migration toward 1 nM *f*MLF was measured, as described. The data are from one experiment that is representative of two independent experiments.

Various essential oils and their components have been reported to inhibit neutrophil chemotaxis [38,39,40]. In the present study, the effects of PGI and its major component compound dillapiole (30.3%) on human neutrophil chemotaxis were evaluated. Pretreatment with PGI dose-dependently inhibited *f*MLF-induced neutrophil chemotaxis (IC_50_ = 10.5 ± 3.3 μg/mL) (Figure 4A). Likewise, pretreatment with dillapiole also inhibited *f*MLF-induced human neutrophil chemotaxis (IC_50_ = 91.3 ± 22.2 µM) (Figure 4B). Because [Ca^2+^]_i_ is involved in neutrophil chemotaxis [9], the inhibitory effect of dillapiole on neutrophil chemotaxis is consistent with its primary effect on Ca^2+^ flux.

**Figure 5:**
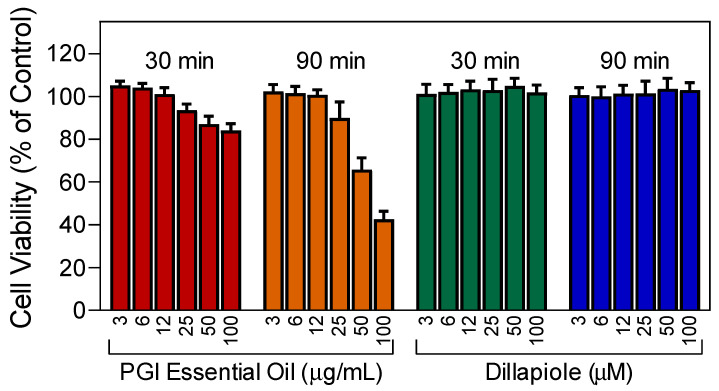
Cytotoxicity of the essential oils from *P. gairdneri* (PGI) and dillapiole. Human neutrophils were preincubated with the indicated concentrations of the essential oils or pure dillapiole for 30 min or 90 min, and cell viability was analyzed, as described. Values are the mean ± SD of triplicate samples from one experiment that is representative of three independent experiments with similar results.

To evaluate the toxicity of essential oils from *P. gairdneri* and dillapiole, we incubated neutrophils with PGI (up to 100 µg/mL) and pure dillapiole at various concentrations (up to 100 µM) and evaluated cell viability. As shown in Figure 5, PGI had little to no cytotoxicity during a 30-min incubation period but was cytotoxic during a 90-min incubation period at concentrations of 50 and 100 µg/mL. Note that the inhibitory effects of PGI on neutrophil functional activity were found at much lower concentrations (<10 µg/mL). Dillapiole had no neutrophil cytotoxicity at all concentrations and times tested (Figure 5).

**Figure 6:**
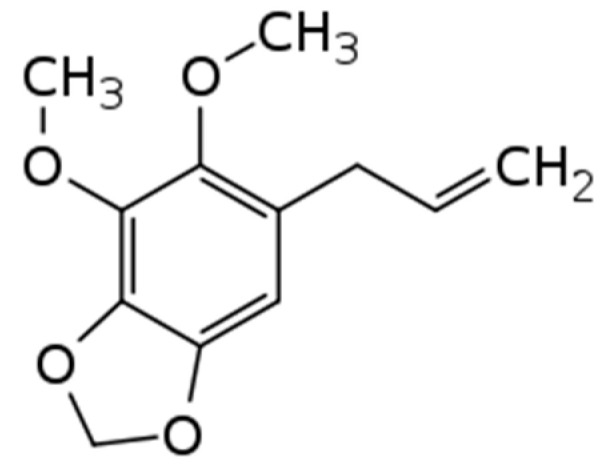
Chemical structure of dillapiole.

This is the first report on the inhibitory effects of dillapiole on human neutrophil activation (Table 4). Dillapiole (see chemical structure in Figure 6) is a phenylpropanoid found in abundance in essential oils from *Piper* species, *Deverra triradiata* Hochst. ex Boiss, and in the early developmental stages of dill (*Anethum graveolens* L.) [41,42,43,44]. It has been reported to exhibit bactericidal [45], fungicidal [46], antileishmanial [47], and gastroprotective activity [48]. Interestingly, dillapiole has also been reported to have anti-inflammatory activity in a carrageenan-induced rat paw edema model [49] and broad cytotoxic effects against a variety of tumor cells [50].

**Figure 7:**
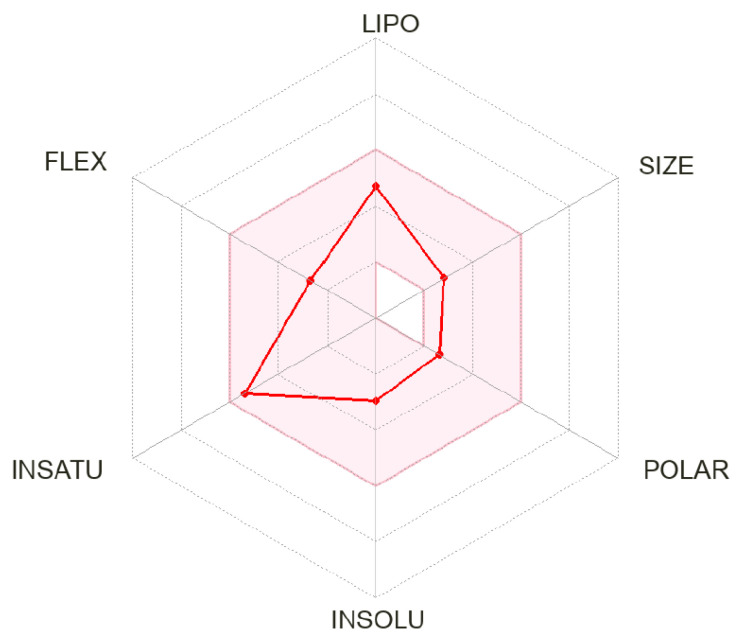
Bioavailability radar plot of dillapiole. The plot depicts the LIPO (lipophilicity), SIZE (molecular weight), POLAR (polarity), INSOLU (insolubility), INSATU (unsaturation), and FLEX (rotatable bond flexibility) parameters.

To further characterize dillapiole, we calculated the most important physico-chemical and ADME parameters of this compound using SwissADME [51] and found that it would be predicted to permeate the blood–brain barrier (BBB) (Table 5). According to the radar plot of the main characteristics, the ADME data for dillapiole predict that it would also exhibit high bioavailability (Figure 7).

One of the issues noted for this research is that DMSO was required for solubilizing our samples, which may be problematic in the development of new therapeutics. However, recent studies by Carneiro et al. [52] indicate that nanoemulsions and nanostructured lipid carriers could be used for the delivery of essential oils and dillapiole. Thus, nanocarriers loaded with dillapiole could potentially represent an interesting strategy for developing this compound for the treatment of inflammation.

## 3. Materials and Methods

### 3.1. Materials

Dimethyl sulfoxide (DMSO), *f*MLF, Histopaque 1077, (−)-α-thujone, α/β-thujone, and dillapiole were purchased from Sigma-Aldrich Chemical Co. (St. Louis, MO, USA). Menthone, isomenthone, and pulegone were from Toronto Research Chemicals (North York, ON, Canada). *n*-Hexane was purchased from Merck (Darmstadt, Germany). Fluo-4AM was purchased from Invitrogen (Carlsbad, CA, USA). Hanks’ balanced salt solution (HBSS; 0.137 M NaCl, 5.4 mM KCl, 0.25 mM Na_2_HPO_4_, 0.44 mM KH_2_PO_4_, 4.2 mM NaHCO_3_, 5.56 mM glucose, and 10 mM HEPES, pH 7.4) was purchased from Life Technologies (Grand Island, NY, USA). HBSS without Ca^2+^ and Mg^2+^ is designated as HBSS^–^; HBSS containing 1.3 mM CaCl_2_ and 1.0 mM MgSO_4_ is designated as HBSS^+^.

### 3.2. Essential Oil Extraction

Essential oils were obtained from the air-dried plant material by hydrodistillation using a Clevenger-type apparatus, as previously described **[37]**. We used conditions accepted by the European Pharmacopoeia (European Directorate for the Quality of Medicines, Council of Europe, Strasbourg, France, 2014) to avoid artifacts. Yields of essential oils were calculated based on the amount of air-dried plant material used.

### 3.3. Gas Chromatography (GC-FID) and Gas Chromatography-Mass Spectrometry (GC-MS) Analysis

Stock solutions of the essential oils were prepared in *n*-hexane (10% *w*/*v*), and GC-MS analysis was performed with an Agilent 5975 GC-MSD system (Agilent Technologies, Santa Clara, CA, USA), as reported previously [53]. An Agilent Innowax FSC column (60 m × 0.25 mm, 0.25 μm film thickness) was used with He as the carrier gas (0.8 mL/min). The GC oven temperature was kept at 60 °C for 10 min, increased to 220 °C at a rate of 4 °C/min, kept constant at 220 °C for 10 min, and then increased to 240 °C at a rate of 1 °C/min. The split ratio was adjusted to 40:1, and the injector temperature was 250 °C. MS spectra were monitored at 70 eV with a mass range of 35 to 450 *m*/*z*. GC analysis was performed using an Agilent 6890N GC system. To obtain the same elution order as with GC-MS, the line was split for FID and MS detectors, and a single injection was performed using the same column and operational conditions. The flame ionization detector (FID) temperature was 300 °C. The essential oil components were identified by co-injection with standards (whenever possible), which were purchased from commercial sources or isolated from natural sources. In addition, compound identities were confirmed by comparison of their mass spectra with those in the Wiley GC/MS Library (Wiley, NY, USA), MassFinder software 4.0 (Dr. Hochmuth Scientific Consulting, Hamburg, Germany), Adams Library, and NIST Library. Confirmation was also achieved using the in-house “Başer Library of Essential Oil Constituents” database, obtained from chromatographic runs of pure compounds performed with the same equipment and conditions. A C_8_–C_40_ *n*-alkane standard solution (Fluka, Buchs, Switzerland) was used to spike the samples for the determination of relative retention indices (RRI). Relative percentage amounts of the separated compounds were calculated from the FID chromatograms.

### 3.4. Sample Preparation for Biological Studies

Stock solutions of the essential oils and pure compounds were prepared in DMSO (10 mg/mL and 10 mM, respectively) for biological evaluation and stored at −20 °C. For dose-response analysis, all dilutions of the essential oils and pure compounds were in DMSO. The final concentration of DMSO in cell media was 1%.

### 3.5. Human Neutrophil Isolation

Human neutrophils were isolated from blood that was collected from healthy donors in accordance with a protocol approved by the Institutional Review Board at Montana State University (Protocol #2022-168). Neutrophils were purified from the blood using dextran sedimentation, followed by Histopaque 1077 gradient separation and hypotonic lysis of red blood cells, as described previously [54]. Neutrophil preparations were routinely >95% pure, as determined by light microscopy, and >98% viable, as determined by trypan blue exclusion.

### 3.6. Ca^2+^ Mobilization Assay

Changes in intracellular Ca^2+^ concentrations ([Ca^2+^]_i_) were measured with a FlexStation 3 scanning fluorometer (Molecular Devices, Sunnyvale, CA, USA), as described previously [53]. Briefly, human neutrophils were suspended in HBSS^-^, loaded with Fluo-4AM at a final concentration of 1.25 μg/mL, and incubated for 30 min in the dark at 37 °C. After dye loading, the cells were washed with HBSS^-^, resuspended in HBSS^+^, separated into aliquots, and loaded into the wells of flat-bottom, half-area well black microtiter plates (2 × 10^5^ cells/well). To measure the direct effects of test compound or pure essential oils on Ca^2+^ flux, the compound/oil was added to the wells (final concentration of DMSO was 1%), and changes in fluorescence were monitored (λ_ex_ = 485 nm, λ_em_ = 538 nm) every 5 s for 240 s at room temperature after addition of the test compound or control agonist for comparison. To evaluate the inhibitory effects of the compounds on Ca^2+^ flux, the compound/oil was added to the wells (the final concentration of DMSO was 1%). The samples were preincubated for 10 min, followed by the addition of 5 nM *f*MLF. The maximum change in fluorescence, expressed in arbitrary units over baseline, was used to determine the agonist response. Responses were normalized to the response induced by 5 nM *f*MLF alone without pretreatment, and these responses were assigned as 100%. Curve fitting (at least five or six points) and calculation of median effective concentration values (EC_50_ or IC_50_) were performed by nonlinear regression analysis of the dose–response curves generated using Prism 9 (GraphPad Software, Inc., San Diego, CA, USA).

### 3.7. Chemotaxis Assay

Human neutrophils were resuspended in HBSS^+^ containing 2% (*v*/*v*) heat-inactivated FBS (2 × 10^6^ cells/mL), and chemotaxis was analyzed in 96-well ChemoTx#105-5 chemotaxis chambers (Neuroprobe, Gaithersburg, MD, USA). In brief, neutrophils were preincubated with the indicated concentrations of the test sample (essential oil or pure compound) or DMSO (1% final concentration) for 30 min at room temperature and added to the upper wells of the ChemoTx chemotaxis chambers (40 × 10^3^ cells/well). The lower wells were loaded with 30 µL of HBSS^+^ containing 2% (*v*/*v*) heat-inactivated FBS, the indicated concentrations of the test sample or control DMSO, and 1 nM *f*MLF as the chemoattractant. Three lower wells were reserved for background controls (DMSO-treated cells in the upper wells and DMSO without *f*MLF in the lower wells). Neutrophils were added to the upper wells and allowed to migrate through the 5.0-µm pore polycarbonate membrane filter for 60 min at 37 °C and 5% CO_2_. The number of migrated cells was determined by measuring ATP in lysates of transmigrated cells using a luminescence-based assay (CellTiter-Glo; Promega, Madison, WI, USA), and luminescence measurements were converted to absolute cell numbers by comparison of the values with standard curves obtained with known numbers of neutrophils. Curve fitting (at least eight to nine points) and calculation of median effective concentration values (IC_50_) were performed by nonlinear regression analysis of the dose–response curves generated using GraphPad Prism 9.

### 3.8. Cytotoxicity Assay

Cytotoxicity of essential oils and pure compounds in human neutrophils was analyzed using a CellTiter-Glo Luminescent Cell Viability Assay Kit (Promega), according to the manufacturer’s protocol. Briefly, human neutrophils were incubated at a density of 10^4^ cells/well with different concentrations of essential oils or compounds (the final concentration of DMSO was 1%) for 90 min at 37 °C and 5% CO_2_. Following treatment, the substrate was added to the cells, and the samples were analyzed with a Fluoroscan Ascent FL microplate reader.

## 4. Conclusions

Analysis of the composition of essential oils extracted from *A. urticifolia*, *A. millefolium*, and *P. gairdneri* collected in Montana subalpine meadows showed that the main components of essential oils obtained from *A. urticifolia* were menthone, isomenthone, pulegone, and limonene; whereas essential oils obtained from *A. millefolium* were high in α-thujone and β-thujone, 1,8-cineole, camphor, sabinene, guaia-3,9-dien-11-ol, and terpinen-4-ol; and essential oils obtained from *P. gairdneri* contained high amounts of dillapiole, *p*-cymen-8-ol, terpinolene, 4-hydroxy-4-methyl-cyclohex-2-enone, and γ-terpinene. Essential oils from these plants inhibited [Ca^2+^]_i_ in human neutrophils, with varying potency. The biological effects of *A. urticifolia* and *A. millefolium* essential oils might be attributable primarily to previously reported active constituents, including bornyl acetate, borneol, germacrene D, and nerolidol. Dillapiole, which was present at high levels in essential oils of *P. gairdneri*, inhibited [Ca^2+^]_i_ in neutrophils and chemotaxis. Thus, dillapiole is likely one of the main active components in these essential oils. Given the critical role of neutrophils in inflammation, these data support the possibility that dillapiole or its structural analogs could be considered in the development of new anti-inflammatory agents. To verify the key targets responsible for the immunomodulatory effects of dillapiole, further experimental investigation is needed.

## Figures and Tables

**Table 1:** Location, date of collection of the plant material, and distillation yields of essential oils.

Plant	Location	Latitude (N)	Longitude (E)	Altitude (m)	Plant Material	Date of Collection	Yield (%)
*A. urticifolia*	Hyalite Canyon, Bozeman, MT, USA	45.48990°	111.00091°	2272	leaves/flowers	07/2021	0.2/0.5
*A. millefolium*	Hyalite Canyon, Bozeman, MT, USA	45.48346°	110.97882°	2042	inflorescence	07/2021	0.2
*P. gairdneri*	Hyalite Canyon, Bozeman, MT, USA	45.49671°	110.98859°	1978	inflorescence	07/2021	1.9

**Table 2:** Composition of essential oils isolated from *A. urticifolia* (AUF and AUL), *A. millefolium* (AMI), and *P. gairdneri* (PGI).

RRI	Compound	AUF	AUL	AMI	PGI	RRI	Compound	AUF	AUL	AMI	PGI
1032	α-Pinene	t	t	1.2	0.2	1704	γ-Muurolene		t		
1035	α-Thujene	t	t	t	t	1706	α-Terpineol		t	1.9	1.1
1048	2-Methyl-3-buten-2-ol	t	t			1715	γ-Terpineol				t
1076	Camphene			2.3	t	1719	Borneol			1.2	
1093	Hexanal	t		t		1720	*trans*-Sabinol			0.2	
1118	β-Pinene	t	t	1.9	t	1726	Germacrene D	0.1		0.1	
1132	Sabinene	0.1	0.1	7.0	0.1	1748	Piperitone	0.1	0.7		
1136	Isoamyl acetate	t	0.4			1751	Carvone	0.1	0.2		t
1174	Myrcene	0.4	0.4	t	0.2	1773	δ-Cadinene			0.2	
1176	α-Phellandrene			t	0.1	1797	*p*-Methyl acetophenone	t			0.6
1185	Isobutyl 2-methyl butyrate		t	t		1802	Cumin aldehyde			0.2	
1188	α-Terpinene			0.2	t	1811	*p*-Mentha-1,3-dien-7-al				0.4
1195	Dehydro-1,8-cineole	t		0.2		1814	*p*-Mentha-1,5-dien-7-ol			0.7	
1203	2-Methyl butyl isobutyrate			t		1838	2-Phenylethyl acetate	0.1	0.2		
1203	Limonene	4.2	6.2	0.2	1.3	1845	*trans*-Carveol	t			
1213	1,8-Cineole	t	t	17.0		1849	Pulegone epoxide		0.4		
1218	β-Phellandrene	0.1	0.1		0.5	1864	*p*-Cymen-8-ol	t			14.1
1225	(*Z*)-3-Hexenal	t	0.2			1865	Isopiperitenone	0.5	0.1		
1244	Amyl furan			t		1877	TMMT	1.1	0.8		
1246	(*Z*)-β-Ocimene	0.1	0.1	t	0.4	1894	CMMT	0.5	0.4		
1255	γ-Terpinene	t		0.4	2.4	1898	1,11-Oxidocalamenene			0.3	
1266	(*E*)-β-Ocimene	0.5	0.3		0.3	1949	Piperitenone	1.6	0.8		
1266	3-Octanone	t				1969	*cis*-Jasmone		0.2		
1280	*p*-Cymene	t		0.7	1.1	1998	8,9-dehydrothymol	0.1	t		
1285	Isoamyl isovalerate	t		t		2008	Caryophyllene oxide	0.3	0.4	0.5	
1286	MBMB			0.1		2016	Isoamyl phenyl acetate		0.1		
1290	Terpinolene	t		0.1	12.0	2045	Carotol				0.2
1384	α-Pinene oxide	0.1				2050	(*E*)-Nerolidol			0.2	
1386	Octenyl acetate	t				2068	Hexahydro-farnesyl acetone	0.1			
1400	Nonanal			t		2074	Caryophylla-2(12),6(13)-dien-5-one			0.3	
1408	1,3,8-*p*-Menthatriene				0.1	2094	*p*-Cresol				0.2
1413	Rose furan	t				2096	Elemol			1.7	
1437	α-Thujone			17.1	0.3	2096	(*E*)-Methyl cinnamate				0.3
1443	2,5- Dimethylstyrene	t				2100	Heneicosane			0.3	
1451	β-Thujone	0.1		14.9		2103	Guaiol			0.1	
1452	α,*p*-Dimethylstyrene	t			0.6	2113	Cumin alcohol			0.3	
1452	1-Octen-3-ol	t				2115	4-Hydroxy-4-methyl-cyclohex-2-enone				6.2
1458	*cis*-1,2-Limonene epoxide	t				2144	Spathulenol	0.1	0.2		
1474	*trans*-Sabinene hydrate			0.3		2181	Isothymol	0.3			
1475	Menthone	2.7	25.7			2183	γ-Decalactone				0.6
1497	α-Copaene			0.2		2184	*cis-p*-Menth-3-en-1,2-diol				0.4
1497	Menthofuran	0.1				2185	γ-Eudesmol			0.9	
1503	Isomenthone	2.6	29.1			2192	Nonanoic acid			0.1	
1532	Camphor			13.0		2195	Fokienol			0.1	
1541	Benzaldehyde	0.2	0.4			2209	T-Muurolol		t		
1553	Linalool			0.3		2221	Isocarvacrol				0.3
1556	*cis*-Sabinene hydrate			0.2		2228	Eremoligenol			0.1	
1571	*trans-p*-Menth-2-en-1-ol			0.1		2245	Elemicine				1.2
1583	*cis*-Isopulegone	0.5	0.2			2250	α-Eudesmol			0.5	
1590	Bornyl acetate			1.7		2250	Fukinanolide				0.6
1598	*trans*-Isopulegone	0.5				2255	α-Cadinol		t		
1611	Terpinen-4-ol			2.5	0.4	2257	β-Eudesmol			1.1	
1612	β-Caryophyllene	0.5	0.3	0.3	0.1	2272	Copaborneol			t	
1618	Camphene hydrate		0.2			2290	Guaia-3,9-dien-11-ol			3.2	
1626	MMO			t		2296	Myristicine				0.1
1638	*cis-p*-Menth-2-en-1-ol			0.1		2303	Menthofurolactone	0.2	t		
1639	*trans-p*-Mentha-2,8-dien-1-ol	0.5	0.6			2316	Caryophylladienol I			0.6	
1642	Thuj-3-en10-al			0.2		2324	Caryophylladienol II			0.2	
1648	Myrtenal			0.6		2368	Eudesma-4(15),7-diene-1-β-ol		t		
1651	Sabinaketone			0.1		2384	Dillapiole				30.3
1658	Sabinyl acetate		0.1	0.2		2420	2-Methyl isoborneol *				1.1
1662	Pulegone	78.9	28.8		0.1	2622	Phytol		t		
1678	*cis-p*-Mentha-2,8-dien-1-ol		0.5			2655	Benzyl benzoate				1.9
1682	δ-Terpineol			0.6		2758	Artedouglasia oxide B				0.6
1690	Cryptone	0.1	0.3		0.2						

The data are presented as relative % calculated from flame ionization detector data for each component identified. RRI, relative retention index calculated based on retention of *n*-alkanes. Trace amounts (t) were present at <0.1%. * Identified tentatively using the Wiley and MassFinder mass spectra libraries and published RRI. All other compounds were identified by comparison with co-injected standards. Abbreviations: AUF, essential oil from flowers of *A. urticifolia*; AUL, essential oil from leaves of *A. urticifolia*; AMI, essential oil from inflorescences of *A. millefolium*; PGI, essential oil from inflorescences of *P. gairdneri*. MMO, 2-methyl-6-methylene-3,7-octadien-2-ol; MBMB, 2-methyl butyl 2-methyl butyrate; TMMT, *trans*-*p*-mentha-8-methylthio-3-one; CMMT, *cis*-p-mentha-8-methylthio-3-one.

**Table 3:** Summary of the chemical composition (%) of essential oils from *A. urticifolia*, *A. millefolium*, and *P. gairdneri*.

Compounds	AUF	AUL	AMI	PGI
Monoterpene hydrocarbons	5.4	7.2	14.0	19.3
Oxygenated monoterpenes	90.6	88.7	73.6	18.4
Sesquiterpene hydrocarbons	0.6	0.3	0.8	0.1
Oxygenated sesquiterpenes	0.4	0.6	9.8	1.4
Oxygenated diterpenes		t		
Phenylpropanoids				31.6
Substituted cyclohexanones				6.2
Others	0.4	1.7	0.5	3.6
Total	97.4	98.5	98.2	80.6

Abbreviations: AUF, essential oil from flowers of *A. urticifolia*; AUL, essential oil from leaves of *A. urticifolia*; AMI, essential oil from inflorescences of *A. millefolium*; PGI, essential oil from inflorescences of *P. gairdneri*.

**Table 4:** Effect of essential oils and their selected component compounds on Ca^2+^ flux in human neutrophils.

Essential Oil or Pure Compound	Direct Activation	Inhibition of *f*MLF-Induced Response ^a^
	EC_50_ (µg/mL); (Efficacy, %)	IC_50_ (µg/mL)
AUF	28.5 ± 2.1 (130)	43.0 ± 2.8
AUL	43.5 ± 6.2 (150)	25.0 ± 4.2
AMI	41.5 ± 0.7 (140)	24.5 ± 2.1
PGI	30.6 ± 4.2 (70)	4.3 ± 2.2
	**EC_50_ (µM); (Efficacy, %)**	**IC_50_ (µM)**
α-Thujene	N.A.	N.A.
α/β-Thujone	N.A.	N.A.
Menthone	N.A.	N.A.
Isomenthone	N.A.	N.A.
Pulegone	N.A.	N.A.
Dillapiole	19.3 ± 1.4 (65)	13.9 ± 4.2

^a^ Inhibition of neutrophil Ca^2+^ flux induced by 5 nM *f*MLF. N.A.: no activity was observed, even at the highest concentration tested (50 µM). EC_50_ and IC_50_ values are presented as the mean ± S.D. of three independent experiments. Efficacy is the maximum response to an essential oil (or compound) compared to that induced by control 5 nM *f*MLF (100%). AUF, essential oil from flowers of *A. urticifolia*; AUL, essential oil from leaves of *A. urticifolia*; AMI, essential oil from inflorescences of *A. millefolium*; PGI, essential oil from inflorescences of *P. gairdneri*.

**Table 5:** Predicted physicochemical properties of dillapiole according to SwissADME results.

Molecular Descriptor	Property
Formula	C_12_H_14_O_4_
M.W.	222.24
Heavy atoms	16
Fraction Csp^3^	0.33
Rotatable bonds	4
H-bond acceptors	4
H-bond donors	0
MR	59.59
tPSA	36.92
iLogP	2.82
BBB permeation	Yes

Abbreviations: M.W., molecular weight (g/mol); MR, molar refractivity; tPSA, topological polar surface area (Å^2^); iLogP, lipophilicity; BBB, blood–brain barrier.

## Data Availability

The data that support the findings of this study are available from the authors upon reasonable request.

## References

[1] Moerman D.E. (2009). Native American Medicinal Plants: An Ethnobotanical Dictionary.

[2] Chandler R.F., Hooper S.N., Harvey M.J. (1982). Ethnobotany and phytochemistry of yarrow, *Achillea millefolium*, Compositae. Econ. Bot..

[3] Ali S.I., Gopalakrishnan B., Venkatesalu V. (2017). Pharmacognosy, phytochemistry and pharmacological properties of *Achillea millefolium* L.: A review. Phytother. Res..

[4] Borrelli F., Romano B., Fasolino I., Tagliatatela-Scafati O., Aprea G., Capasso R., Capasso F., Bottazzi E.C., Izzo A.A. (2012). Prokinetic effect of a standardized yarrow (*Achillea millefolium*) extract and its constituent choline: Studies in the mouse and human stomach. Neurogastroenterol. Motil..

[5] Sandner G., Heckmann M., Weghuber J. (2020). Immunomodulatory activities of selected essential oils. Biomolecules.

[6] Gandhi G.R., Vasconcelos A.B.S., Haran G.H., Calisto V., Jothi G., Quintans J.S.S., Cuevas L.E., Narain N., Junior L.J.Q., Cipolotti R. (2020). Essential oils and its bioactive compounds modulating cytokines: A systematic review on anti-asthmatic and immunomodulatory properties. Phytomedicine.

[7] Valdivieso-Ugarte M., Gomez-Llorente C., Plaza-Diaz J., Gil A. (2019). Antimicrobial, antioxidant, and immunomodulatory properties of essential oils: A systematic review. Nutrients.

[8] Beutler B. (2004). Innate immunity: An overview. Mol. Immunol..

[9] Bokoch G.M. (1995). Chemoattractant signaling and leukocyte activation. Blood.

[10] Schepetkin I.A., Ozek G., Ozek T., Kirpotina L.N., Kokorina P.I., Khlebnikov A.I., Quinn M.T. (2022). Neutrophil Immunomodulatory activity of nerolidol, a major component of essential oils from *Populus balsamifera* buds and propolis. Plants.

[11] Schepetkin I.A., Ozek G., Ozek T., Kirpotina L.N., Khlebnikov A.I., Quinn M.T. (2022). Neutrophil immunomodulatory activity of (−)-borneol, a major component of essential oils extracted from *Grindelia squarrosa*. Molecules.

[12] Schepetkin I.A., Ozek G., Ozek T., Kirpotina L.N., Khlebnikov A.I., Quinn M.T. (2021). Chemical composition and immunomodulatory activity of essential oils from *Rhododendron albiflorum*. Molecules.

[13] Schepetkin I.A., Ozek G., Ozek T., Kirpotina L.N., Khlebnikov A.I., Klein R.A., Quinn M.T. (2022). Neutrophil immunomodulatory activity of farnesene, a component of *Artemisia dracunculus* essential oils. Pharmaceuticals.

[14] Ozek G., Schepetkin I.A., Yermagambetova M., Ozek T., Kirpotina L.N., Almerekova S.S., Abugalieva S.I., Khlebnikov A.I., Quinn M.T. (2021). Innate immunomodulatory activity of cedrol, a component of essential oils isolated from *Juniperus* species. Molecules.

[15] Tadic V., Arsic I., Zvezdanovic J., Zugic A., Cvetkovic D., Pavkov S. (2017). The estimation of the traditionally used yarrow (*Achillea millefolium* L. Asteraceae) oil extracts with anti-inflamatory potential in topical application. J. Ethnopharmacol..

[16] Bogdan A., Colţun M. (2020). *Agastache urticifolia* (Benth.) Kuntze–aromatic plant introduced and researched in the Botanical Garden. Rev. Bot..

[17] Wilson T.M., Davis A., Sonstrom R.E., Neill J.L., Ziebarth E.A., Poulson A., Carlson R.E. (2023). Essential oil composition and enantioselective profile of *Agastache urticifolia* (Lamiaceae) and *Monardella odoratissima* (Lamiaceae) from Utah. Molecules.

[18] Zielinska S., Matkowski A. (2014). Phytochemistry and bioactivity of aromatic and medicinal plants from the genus *Agastache* (Lamiaceae). Phytochem. Rev..

[19] Bogdan A., Colţun M., Gille E., Necula R.D., Grigoraş V. The biology and the chemical composition of the essential oil of the specie Agastache urticifolia (Benth). Kuntze. Proceedings of the V International Scientific and Practical Conference within the Framework of the VI Scientific Forum “Science Week in Kruty”.

[20] Myadelets M.A., Vorobyeva T.A., Domrachev D.V. (2013). Composition of the essential oils of some species belonging to genus *Agastache clayton* ex Gronov (Lamiaceae) cultivated under the conditions of the Middle Ural. Chem. Sustain. Dev..

[21] Viljoen A.M., Moolla A., van Vuuren S.F., van Zyl R.L., Baser K.H.C., Demirci B., Ozek T., Trinder-Smith T.H. (2006). The biological activity and essential oil composition of 17 *Agathosma* (Rutaceae) species. J. Essent. Oil Res..

[22] Alan S., Kurkcuoglu M., Baser K.H.C. (2011). Composition of essential oils of *Calamintha nepeta* (L.) Savi Subsp nepeta and *Calamintha nepeta* (L.) Savi Subsp glandulosa (Req.) PW Ball. Asian J. Chem..

[23] Kaiser R., Lamparsky D., Schudel P. (1975). Analysis of Buchu leaf oil. J. Agr. Food Chem..

[24] Orav A., Arak E., Raal A. (2006). Phytochemical analysis of the essential oil of *Achillea millefolium* L. from various European countries. Nat. Prod. Res..

[25] Falconieri D., Piras A., Porcedda S., Marongiu B., Goncalves M.J., Cabral C., Cavaleiro C., Salgueiro L. (2011). Chemical composition and biological activity of the volatile extracts of *Achillea millefolium*. Nat Prod Commun.

[26] Tampe J., Parra L., Huaiquil K., Mutis A., Quiroz A. (2015). Repellent effect and metabolite volatile profile of the essential oil of *Achillea millefolium* against *Aegorhinus nodipennis* (Hope) (Coleoptera: Curculionidae). Neotrop. Entomol..

[27] Toplan G.G., Taskin T., Iscan G., Goger F., Kurkcuoglu M., Civas A., Ecevit-Genc G., Mat A., Baser K.H.C. (2022). Comparative studies on essential oil and phenolic content with in vitro antioxidant, anticholinesterase, antimicrobial activities of *Achillea biebersteinii* Afan. and *A. millefolium* subsp. millefolium Afan. L. growing in Eastern Turkey. Molecules.

[28] Acimovic M., Zoric M., Zheljazkov V.D., Pezo L., Cabarkapa I., Jeremic J.S., Cvetkovic M. (2020). Chemical characterization and antibacterial activity of essential oil of medicinal plants from Eastern Serbia. Molecules.

[29] El-Kalamouni C., Venskutonis P.R., Zebib B., Merah O., Raynaud C., Talou T. (2017). Antioxidant and antimicrobial activities of the essential oil of *Achillea millefolium* L. grown in France. Medicines.

[30] Millefolii Herba Y. (2016). European Pharmacopoeia. European Directorate for the Quality of Medicines & HealthCare of the Council of Europe (EDQM).

[31] Raal A., Orav A., Arak E. (2012). Essential oil content and composition in commercial *Achillea millefolium* L. herbs from different countries. J. Essent. Oil Bear. Plants.

[32] Rauchensteiner F., Nejati S., Saukel J. (2004). The *Achillea millefolium* group (Asteraceae) in Middle Europe and the Balkans: A diverse source for the crude drug *Herba Millefolii*. J. Trad. Medi..

[33] Schepetkin I.A., Ozek G., Ozek T., Kirpotina L.N., Khlebnikov A.I., Quinn M.T. (2020). Chemical composition and immunomodulatory activity of *Hypericum perforatum* essential oils. Biomolecules.

[34] Naya Y., Nagahama Y., Kotake M. (1978). Volatile components of *Ledum palustre* Var Nipponicum Et Yesoense. Heterocycles.

[35] Madyastha K.M., Raj C.P. (2002). Stereoselective hydroxylation of 4-methyl-2-cyclohexenone in rats: Its relevance to R-(+)-pulegone-mediated hepatotoxicity. Biochem. Biophys. Res. Comm..

[36] Schepetkin I.A., Kushnarenko S.V., Ozek G., Kirpotina L.N., Sinharoy P., Utegenova G.A., Abidkulova K.T., Ozek T., Baser K.H., Kovrizhina A.R. (2016). Modulation of Human neutrophil responses by the essential oils from *Ferula akitschkensis* and their constituents. J. Agric. Food Chem..

[37] Schepetkin I.A., Kushnarenko S.V., Ozek G., Kirpotina L.N., Utegenova G.A., Kotukhov Y.A., Danilova A.N., Ozek T., Baser K.H., Quinn M.T. (2015). Inhibition of human neutrophil responses by the essential oil of *Artemisia kotuchovii* and its constituents. J. Agric. Food Chem..

[38] Kreutz T., Carneiro S.B., Soares K.D., Limberger R.P., Apel M.A., Veiga V.F., Koester L.S. (2021). *Aniba canelilla* (Kunth) Mez essential oil-loaded nanoemulsion: Improved stability of the main constituents and in vitro antichemotactic activity. Ind. Crops Prod..

[39] Maciel A.J., Lacerda C.P., Danielli L.J., Bordignon S.A.L., Fuentefria A.M., Apel M.A. (2019). Antichemotactic and antifungal action of the essential oils from *Cryptocarya aschersoniana*, *Schinus terebinthifolia*, and *Cinnamomum amoenum*. Chem. Biodiver..

[40] Soares K.D., Bordignon S.A.L., Apel M.A. (2022). Chemical composition and anti-inflammatory activity of the essential oils of *Piper gaudichaudianum* and *Piper mikanianum*. J. Ethnopharmacol..

[41] Santana A.I., Vila R., Canigueral S., Gupta M.P. (2016). Chemical composition and biological activity of essential oils from different species of *Piper* from Panama. Planta Med..

[42] Ferreira O.O., Cruz J.N., de Moraes A.A.B., Franco C.D.P., Lima R.R., Dos Anjos T.O., Siqueira G.M., do Nascimento L.D., Cascaes M.M., de Oliveira M.S. (2022). Essential oil of the plants growing in the Brazilian Amazon: Chemical composition, antioxidants, and biological applications. Molecules.

[43] Vila R., Tomi M., Mundina M., Santana A.I., Solis P.N., Arce J.B.L., Iclina J.L.B., Iglesias J., Gupta M.P., Casanova J. (2005). Unusual composition of the essential oils from the leaves of *Piper aduncum*. Flavour Fragr. J..

[44] Guetat A., Abdelwahab A.T., Yahia Y., Rhimi W., Alzahrani A.K., Boulila A., Cafarchia C., Boussaid M. (2022). *Deverra triradiata* Hochst. ex Boiss. from the Northern Region of Saudi Arabia: Essential oil profiling, plant extracts and biological activities. Plants.

[45] Brazao M.A.B., Brazao F.V., Maia J.G.S., Monteiro M.C. (2014). Antibacterial activity of the *Piper aduncum* oil and dillapiole, its main constituent, against multidrug-resistant strains. Bol. Latinoam. Caribe Plantas.

[46] de Almeida R.R.P., Souto R.N.P., Bastos C.N., da Silva M.H.L., Maia J.G.S. (2009). Chemical variation in *Piper aduncum* and biological properties of its dillapiole-rich essential oil. Chem. Biodiver..

[47] Parise R., Pasqualoto K.F.M., Magri F.M.M., Ferreira A.K., da Silva B.A.V.G., Damiao M.C.F.C.B., Tavares M.T., Azevedo R.A., Auada A.V.V., Polli M.C. (2012). Dillapiole as antileishmanial agent: Discovery, cytotoxic activity and preliminary SAR studies of dillapiole analogues. Arch. Pharm..

[48] Rojas-Martinez R., Arrieta J., Cruz-Antonio L., Arrieta-Baez D., Velazquez-Mendez A.M., Sanchez-Mendoza M.E. (2013). Dillapiole, isolated from *Peperomia pellucida*, shows gastroprotector activity against ethanol-induced gastric lesions in Wistar rats. Molecules.

[49] Parise R., Pastrello M., Camerlingo C.E.P., Silva G.J., Agostinho L.A., de Souza T., Magri F.M.M., Ribeiro R.R., Brandt C.A., Polli M.C. (2011). The anti-inflammatory activity of dillapiole and some semisynthetic analogues. Pharmaceut. Biol..

[50] Ferreira A.K., de-Sa P.L., Pasqualoto K.F.M., de Azevedo R.A., Camara D.A.D., Costa A.S., Figueiredo C.R., Matsuo A.L., Massaoka M.H., Auada A.V.V. (2014). Cytotoxic effects of dillapiole on MDA-MB-231 cells involve the induction of apoptosis through the mitochondrial pathway by inducing an oxidative stress while altering the cytoskeleton network. Biochimie.

[51] Daina A., Michielin O., Zoete V. (2017). SwissADME: A free web tool to evaluate pharmacokinetics, drug-likeness and medicinal chemistry friendliness of small molecules. Sci. Rep..

[52] Carneiro S.B., Kreutz T., Limberger R.P., Teixeira H.F., da Veiga Júnior V.F., Koester L.S. (2022). *Piper aduncum* essential oil rich in dillapiole: Development of hydrogel-thickened nanoemulsion and nanostructured lipid carrier intended for skin delivery. Pharmaceutics.

[53] Ozek G., Ishmuratova M., Tabanca N., Radwan M.M., Goger F., Ozek T., Wedge D.E., Becnel J.J., Cutler S.J., Can Baser K.H. (2012). One-step multiple component isolation from the oil of *Crinitaria tatarica* (Less.) Sojak by preparative capillary gas chromatography with characterization by spectroscopic and spectrometric techniques and evaluation of biological activity. J. Sep. Sci..

[54] Schepetkin I.A., Kirpotina L.N., Khlebnikov A.I., Quinn M.T. (2007). High-throughput screening for small-molecule activators of neutrophils: Identification of novel *N*-formyl peptide receptor agonists. Mol. Pharmacol..

